# Integrating breast cancer polygenic risk scores at scale in the WISDOM Study: a national randomized personalized screening trial

**DOI:** 10.1186/s13073-025-01524-7

**Published:** 2025-08-28

**Authors:** Kirkpatrick B. Fergus, Rachel S. Heise, Lisa Madlensky, Allison Fiscalini, Leah Sabacan, Sarah Theiner, Shreya Kapoor, Irene A. Soto, Amie Blanco, Katherine Ross, Deborah Goodman-Gruen, Maren Scheuner, Donglei Hu, Diane Heditsian, Susie Brain, Vignesh A. Arasu, Andrea Kaster, Lisa Chapa, Olufunmilayo I. Olopade, Martin Eklund, Jeffrey A. Tice, Elad Ziv, Laura van ‘t Veer, Laura J. Esserman, Yiwey Shieh, The Athena/WISDOM Network Collaborators and Advocate Partners

**Affiliations:** 1 Department of Surgery, University of California, San Francisco, CA USA; 2 Department of Population Health Sciences, Weill Cornell Medicine, New York, NY USA; 3 Department of Medicine, University of California, San Diego, CA USA; 4 Department of Medicine, Santa Clara Valley Medical Center, San Jose, CA USA; 5 Department of Cancer Genetics and Prevention, Helen Diller Family Comprehensive Cancer Center, University of California, San Francisco, CA USA; 6 Department of Epidemiology and Biostatistics, University of California, Irvine, CA USA; 7 Department of Medicine, University of California, San Francisco, CA USA; 8 Breast Oncology Program, Breast Science Advocacy Core, University of California, San Francisco, CA USA; 9 Kaiser Permanente Division of Research, Pleasanton, CA USA; 10 Sanford Health, Sioux Falls, SD USA; 11 Doctors Hospital at Renaissance, Edinburg, TX USA; 12 Center for Clinical Cancer Genetics and Global Health, Department of Medicine, The University of Chicago, Chicago, IL USA; 13 Department of Medical Epidemiology and Biostatistics, Karolinska Institutet, Stockholm, Sweden; 14 Department of Laboratory Medicine, University of California, San Francisco, CA USA

**Keywords:** Polygenic risk scores, Breast neoplasms, Risk prediction, Translational genetics

## Abstract

**Background:**

The Women Informed to Screen Depending On Measures of risk (WISDOM) Study is the first prospective, population-wide application of personalized breast cancer screening. We aim to demonstrate the feasibility of the study’s novel use of polygenic risk scores (PRSs) to tailor screening, evaluate our strategy for adapting PRSs to diverse populations, and quantify the impact of incorporating PRS on the study’s screening recommendations.

**Methods:**

WISDOM is a randomized, preference-tolerant screening trial in the USA testing the safety and morbidity of risk-based versus annual screening in women aged 40–74 without a prior history of breast cancer. This early report includes participants in the risk-based arm only and compares screening recommendations generated by the Breast Cancer Surveillance Consortium (BCSC) clinical risk model alone versus the BCSC model modified by a PRS (BCSC-PRS). The main outcome of interest is the proportion of participants with a change in screening recommendation after integrating PRS for risk stratification.

**Results:**

In the risk-based arm, 21,631 participants received a PRS. Small but statistically significant differences in the PRS were seen between major racial and ethnic groups (*p* < 0.001), and higher PRS was associated with greater extent of family history (*p* < 0.001) and denser breasts (*p* < 0.001). BCSC-PRS risk estimates changed the screening recommendations for 14% of women aged 40–49 compared to BCSC alone and for 10% of women aged 50–74. Projected net screening encounters at the population level were similar for both age groups.

**Conclusions:**

In a first-in-kind application of PRS to inform breast cancer screening approaches, we demonstrate feasibility for scaled implementation, moderate changes to individual screening recommendations, and minimal projected downstream burden on the healthcare system.

**Trial registration:**

Prospectively registered on ClinicalTrials.gov as NCT02620852 on 12/2/2015.

**Supplementary Information:**

The online version contains supplementary material available at 10.1186/s13073-025-01524-7.

## Background

Breast cancer screening recommendations remain largely unchanged since the advent of screening mammograms in the late twentieth century [1, 2]. Age continues to drive screening recommendations, despite oncology entering a personalized or “precision” era where treatments are increasingly tailored to disease-level characteristics. A major translational gap exists between new breast cancer risk prediction tools—such as genetic testing—and the clinical setting, with little knowledge of how risk should be used to guide decision-making around screening and prevention.

The Women Informed to Screen Depending On Measures of risk (WISDOM) Study was conceived to bridge this gap and provide high-quality evidence on risk-based screening. WISDOM is a randomized trial comparing the efficacy, safety, acceptability, and healthcare value of personalized breast cancer screening versus annual mammography [3]. Participants in the personalized arm undergo clinical risk assessment and genetic testing to detect pathogenic variants (PVs) and construct a polygenic risk score (PRS), which predicts breast cancer risk based on the combined effects of common genetic variants each of small effect. As such, WISDOM represents one of the first population-wide applications of PRS to inform the starting age, interval, and modality of screening [4].

Breast cancer PRSs have been consistently shown to improve the performance of existing risk models [5–7] and can stratify risk on a population level with the top 10th percentile having 2- to fourfold increased risk and the bottom 10th percentile having a 2- to fourfold decreased risk compared with the middle 40–60th percentile [8]. While extensive work has gone into developing and validating PRSs for breast cancer, there have been few prospective studies of using PRS in the clinical setting [9]. The WISDOM Study provides a unique opportunity to examine the implementation of PRS for breast cancer screening and prevention. We report our experience implementing PRS in WISDOM, describe its association with patient characteristics including genetic and non-genetic risk factors, and describe the impact of the PRS on screening recommendations when integrated with an existing clinical risk model.

## Methods

### Population and enrollment

The WISDOM Study (prospectively registered on ClinicalTrials.gov as NCT02620852 on 12/2/2015) is a pragmatic, nationwide randomized trial comparing risk-based to annual breast cancer screening. The two co-primary outcomes are safety (advanced cancer incidence) and morbidity (rates of breast biopsies). Secondary outcomes include rates of stage IIB and clinically detected interval cancers combined; rates of stage IIA cancer; patient acceptability (adherence to recommended screening, anxiety, breast cancer worry); endocrine risk reduction uptake; rates of in situ breast cancer; and rates of recalls, follow-up procedures, and systemic therapy. WISDOM enrolled between the dates of 8/31/2016 and 2/28/2023. The main trial outcomes are currently being assessed, with results expected to be reported in the late fall of 2025.

Eligibility criteria for the overall trial included women aged 40–74 without a history of invasive or in situ breast cancer or prior prophylactic bilateral mastectomy. Participants were recruited from participating health centers throughout the continental USA as well as from large health networks such as University of California Health, Sanford Health, and the Veterans Health Administration system. Recruitment took place entirely online, and methods included referrals, emails, outreach events, and community partnerships. Participants were included in the trial after providing informed consent and completion of an online baseline questionnaire and assignment to a screening arm.

**Fig. 1:**
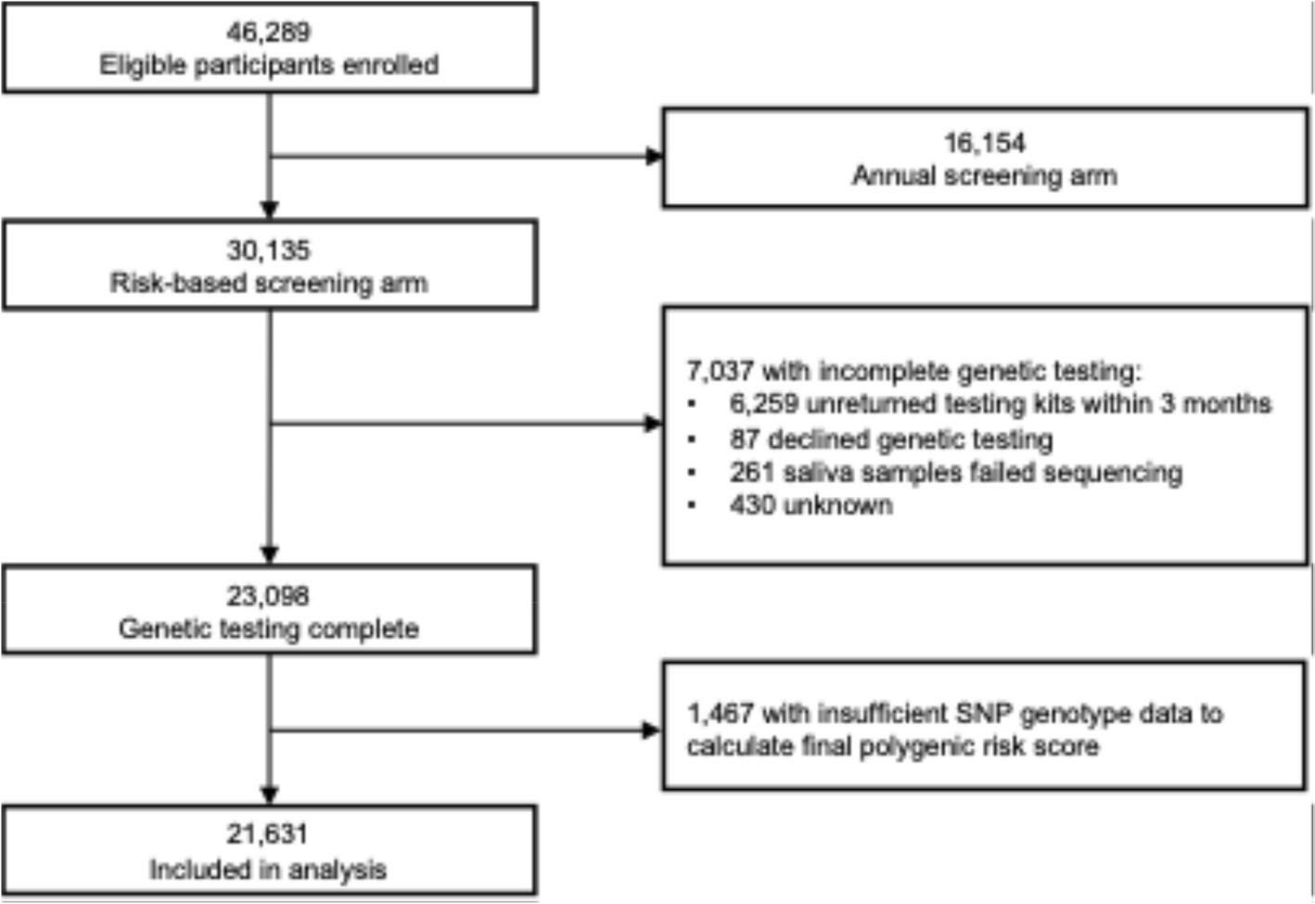
CONSORT diagram. CONSORT diagram for our analysis of a subset of participants from the WISDOM Study. We included participants in the risk-based arm who completed genetic testing and had sufficient single nucleotide polymorphisms genotyped to calculate the full polygenic risk score used in the study

Our main analysis included all participants in the risk-based arm for whom the final version of the trial PRS, effective October 2019, was calculated (Fig. 1). Of 30,135 participants enrolled in the trial who were randomized into or self-selected the risk-based arm, we excluded those who did not successfully complete genetic testing (*n* = 7037). Of the remaining 23,098 participants, 1467 received previous versions of the PRS, which contains fewer SNPs than the final PRS, and were also excluded from the main analysis. Our final analytic cohort included 21,631 participants. All research activities were approved by the Institutional Review Board.

### Ascertainment of participant characteristics and clinical risk factors

We collected demographic and risk factor information using detailed questionnaires at baseline and at yearly follow-ups. Participants self-reported demographic factors such as race, ethnicity, and first- and second-degree family history of cancer. We ascertained breast density, categorized as Breast Imaging Reporting and Data System (BI-RADS) categories a through d, from electronic health records, transmitted results from screening centers, or participant self-report. We calculated the 5-year breast cancer risk based on the Breast Cancer Surveillance Consortium (BCSC) [10] risk model version 2.0, which includes age, race, ethnicity, first-degree relative with breast cancer, pathology of any prior breast biopsy, and BI-RADS density [10].

### Genotyping

We collaborated with Color Health (Burlingame, CA, USA), which performed next-generation sequencing (NGS) of saliva samples for SNP genotyping in tandem with hereditary cancer genetic testing. The NGS protocol has been described previously [11] and is described in detail in the Supplementary Methods.

### PRS construction

Through the adaptive design of WISDOM, we updated the PRS during the trial to reflect the discovery of additional SNPs associated with breast cancer risk. Our initial PRSs contained 75 SNPs [10, 12, 13] without (October 2016–February) and with (March 2018–September 2019) adjustment for race and ethnicity and were similar in composition to those reported in the literature at that time [14]. The final version, implemented in October 2019, expanded the PRS to incorporate SNPs from newly published GWAS [15]. Since we prospectively expanded our targeted SNP panel to include these additional SNPs as available in the targeted sequencing panel, we were unable to retrospectively genotype these SNPs for participants who received earlier versions of the PRS. We constructed separate PRSs for four major groups based on self-reported race and ethnicity, with the number of SNPs in each PRS ranging from 118 to 126 (Table S1; Supplementary Methods). These groups were non-Hispanic (NH) Asian (Asian, Native Hawaiian, Pacific Islander), NH Black, Hispanic (for any participant indicating Hispanic ethnicity, regardless of race), and NH White. For Native American participants and those indicating “other,” “mixed race,” or those who did not report a race, we calculated their PRS using parameters for NH White women given the paucity of GWAS data in these groups. We incorporated population-specific allele frequencies to center the mean PRS to approximately 1 in cancer-free individuals within each respective population (Supplementary Methods) [5, 16]. To generate the BCSC-PRS risk estimate, we combined the PRS with the BCSC 5-year risk estimate in a Bayesian manner to generate the posterior probability of breast cancer, as previously described [5]. The coefficients for the BCSC score have been published [10] and the source code is publicly available at https://tools.bcsc-scc.ucdavis.edu/BC5yearRisk_V2/sourcecode.htm. The statistical code used to generate the PRS and BCSC-PRS estimates can be found at https://github.com/dongleihu/WISDOM-PRS-v3.

### Screening assignment

WISDOM randomizes participants 1:1 to risk-based or annual screening, but is preference-tolerant, meaning participants may choose to forego randomization and self-select their study arm; the latter group is retained as an observational cohort. Participants in the risk-based screening arm undergo multi-gene panel-based testing for PVs and likely PVs in nine moderate or high-penetrance genes, and those found to carry a PV are recommended screening according to established guidelines. Participants testing negative for PVs receive a screening recommendation (Table S2) according to their 5-year breast cancer risk as estimated by the BCSC-PRS risk estimate. Risk estimates are recalculated yearly to reflect new risk factors or changes to the risk stratification algorithm. In this study, we also report the actual year 1 screening assignment given that the final assignment is subject in some cases to manual review by a Screening Review Board (Table S3).

### Statistical analysis

We performed descriptive analyses comparing the mean (standard deviation, SD) and median (interquartile range, IQR) PRS across demographic subgroups. We used Wilcoxon rank sum tests for comparisons between two categories and the Kruskal–Wallis test for comparisons between three or more categories. We assessed correlations between continuous variables using a two-sided Pearson correlation coefficient.

We examined the impact of PRS on screening assignments using cross-tabulations and Sankey plots (flow diagrams) showing the distributions of screening recommendations based on BCSC score only versus based on BCSC-PRS. We examined screening assignments for year 1 (participant’s first year on trial) and determined the screening recommendations using the BCSC score and BCSC-PRS, respectively, by juxtaposing the risk estimate with the trial’s risk thresholds (Table S2) [4]. For these analyses, we excluded PV carriers given that PRS was not used to inform screening recommendations for this group and stratified by two age categories, 40–49 and 50–74.

To determine the impact of PRS on informing screening recommendations, we estimated the percentage of participants with a different screening recommendation according to BCSC-PRS and BCSC alone. To do so, we divided the number of participants with discordant screening recommendations under BCSC-PRS versus BCSC by the total number of participants in the age category. We also compared the projected number of screening examinations per participant for year 1 in the same age categories. As sensitivity analysis, we repeated the screening recommendations and screens per year analysis including all participants with a year 1 assignment regardless of PRS version. Within the women aged 40–49 recommended to start screening in year 1 because of their PRS, we also calculated the percentage that would have been recommended to start screening in years 2–3 due to their BCSC score alone. All statistical analyses were performed using R (version 4.3.0).

## Results

### Demographics and PRS distribution by race

Of 30,135 participants in the risk-based arm, we excluded those who did not successfully complete genetic testing (*n* = 7037) (Fig. 1). The main reason for non-completion was failure to return the testing kit (*n* = 6259). Of the remaining 23,098 participants, 1467 received previous versions of the PRS and were also excluded from the main analysis. Our analytic cohort included 21,631 participants who received the final (October 2019) version of the PRS (Table 1). Participants excluded from our analysis due to missing PRS or earlier PRS version were younger, less likely to be non-Hispanic White, and less likely to have a positive family history of breast cancer, carry a pathogenic variant, or have dense breasts (Table S4).

**Table 1:** Baseline characteristics of WISDOM participants with completed polygenic risk score

Characteristic	*N* = 21,631^a^
Age	53 (45, 62)
Race or ethnicity^b^	
Hispanic	1932 (8.9%)
NH American Indian or Alaska Native	44 (0.2%)
NH Asian/NH Hawaiian, Pacific Islander	1029 (4.8%)
NH Black	969 (4.5%)
NH more than one race/other race/unknown race	875 (4.0%)
NH White	16,782 (78%)
Family history of breast cancer	
No family history	10,433 (48%)
Only second-degree relative	5302 (25%)
Only first-degree relative	3369 (16%)
Both first- and second-degree relative	2527 (12%)
Pathogenic variant carrier	669 (3.1%)
Breast density (BI-RADS category)	
a. Almost entirely fatty	1009 (4.7%)
b. Scattered areas of fibroglandular density	7565 (35%)
c. Heterogeneously dense	8581 (40%)
d. Extremely dense	1876 (8.7%)
Unknown	2600 (12%)

^a^Median (IQR), *n* (%)

^b^*NH* Non-Hispanic

**Fig. 2:**
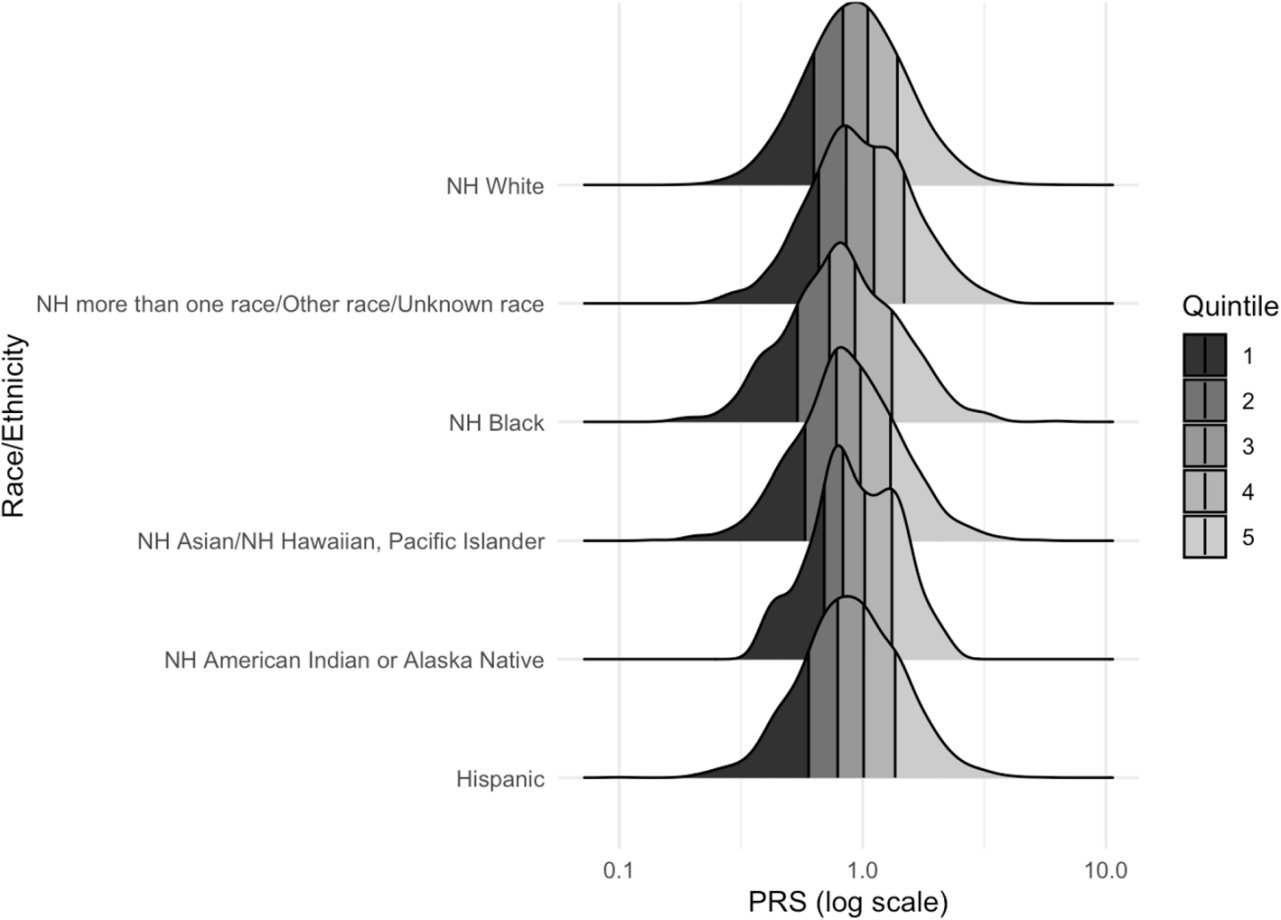
Distribution of PRS by race and ethnicity. Distribution of polygenic risk scores by major race and ethnicity categories used in the WISDOM Study. Vertical lines denote quintile cutoffs for each race and ethnicity group. Pearson’s chi-squared test *p* < 0.001

The overall PRS was right-skewed, with a median (IQR) of 0.92 (0.67–1.28) and mean (SD) of 1.04 (0.54). The mean PRS approximated 1 in the four racial and ethnic groups for whom we calculated separate PRSs, though differences met statistical significance (*p* < 0.001) (Table S5). The PRS value corresponding to the top quintile was similar by groups (Fig. 2), and the overall trial PRS had similar proportions of the major racial groups in the top quintile (Table S6), suggesting that there was minimal systematic overestimation of risk. However, cutoffs for the bottom quintiles of PRS varied by race and ethnicity, suggesting slight underestimation of risk in Asian, Black, and Hispanic participants.

### PRS by family history

Approximately 27% of participants reported a first-degree family history of breast cancer and 36% reported a second-degree history. More extensive family history was associated with higher PRS, with PRS highest in participants with both first- and second-degree family history with a mean (SD) of 1.13 (0.59), and lowest in those with no family history (1.00 (0.51)) (Table 2). Assuming a rank-ordering of these categories of family history, we found evidence of a trend (*p* < 0.001). A sub-analysis found that participants with more relatives with breast cancer had a higher PRS compared with those that had no family history of breast cancer (Table S7).

**Table 2:** Polygenic risk score by extent of family history of breast cancer

	Overall *N* = 21,631	No family history *N* = 10,433	SDR^a^ only *N* = 5302	FDR^b^ only *N* = 3369	Both FDR^a^ and SDR^b^ *N* = 2527	*p* value^c^
PRS^d^						< 0.001
Median (IQR)	0.92 (0.67, 1.28)	0.89 (0.65, 1.23)	0.93 (0.67, 1.28)	0.97 (0.70, 1.34)	1.00 (0.73, 1.39)	
Mean (SD)	1.04 (0.54)	1.00 (0.51)	1.05 (0.56)	1.08 (0.55)	1.13 (0.59)	

^a^*SDR* Second-degree relative with a history of breast cancer

^b^*FDR* First-degree relative with a history of breast cancer

^c^Kruskal-Wallis rank sum test

^d^*PRS* Polygenic risk score, *IQR* Interquartile range, *SD* Standard deviation

### PRS by other clinical risk factors

PRS was positively associated with breast density. Participants with BI-RADS a density had a lower PRS than those with BI-RADS d, mean (SD) of 0.98 (0.48) vs 1.08 (0.57) (Table S8). A test of trend across all BI-RADS categories met statistical significance (*p* < 0.001). We found a weak, but statistically significant, correlation between PRS and the BCSC score, *r*^2^ = 0.0041 (*p* < 0.001) (Fig. S1).

### PRS and screening assignments

#### Screening assignment changes

**Fig. 3:**
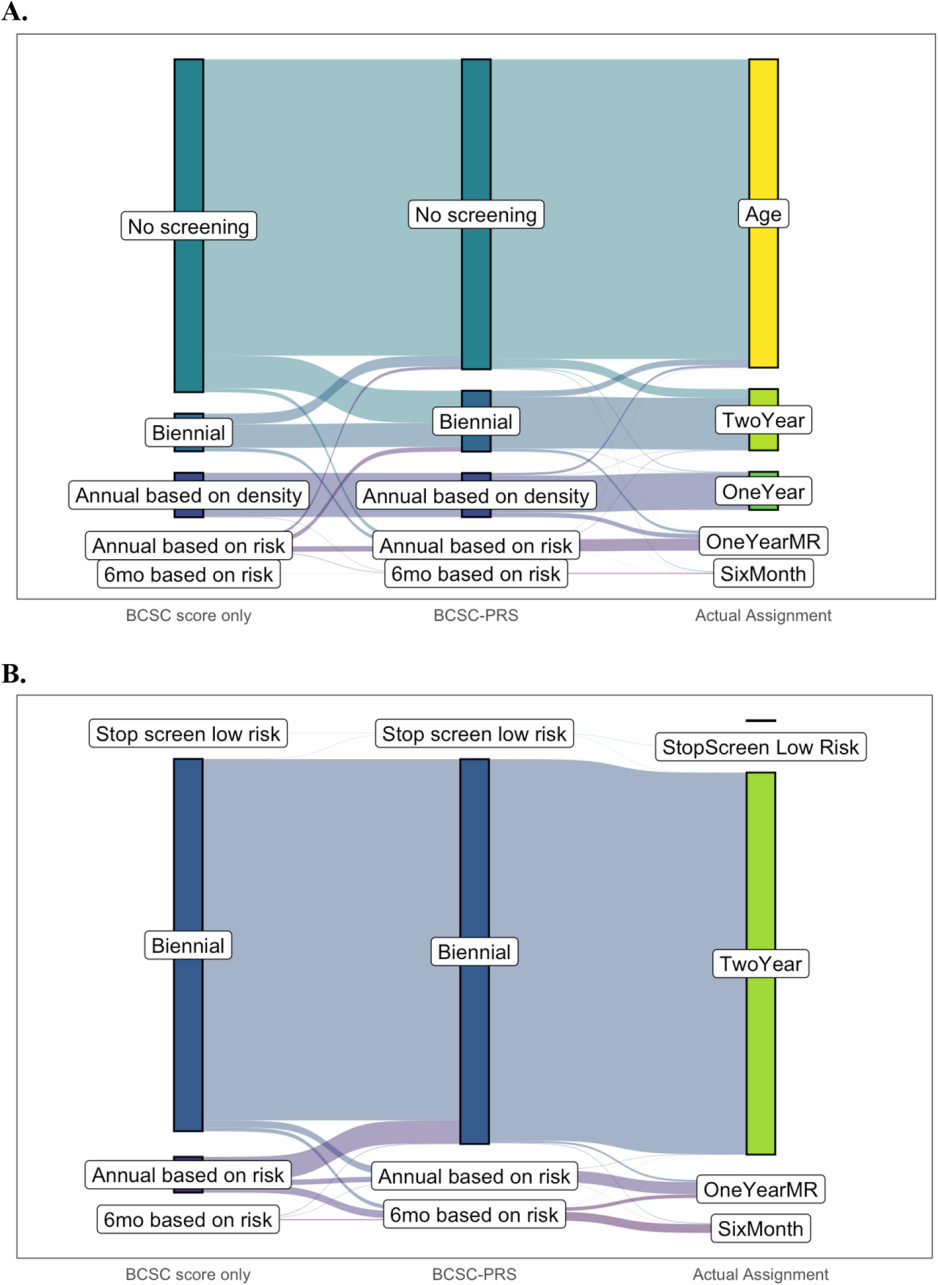
Sankey plots of screening assignment change. Sankey plots for initial (study year 1) participant screening assignments for **A** participants aged 40–49 and **B** participants aged 50–74. The leftmost column of each panel shows screening recommendations based on the Breast Cancer Surveillance Consortium (BCSC) model alone, which include no screening or stop screening, biennial screening (mammogram every other year), annual screening (yearly mammogram) based on risk or density, and screening every 6 months based on risk (alternating mammogram and MRI). The middle column represents screening recommendations based on the BCSC model modified by a polygenic risk score (BCSC-PRS). The rightmost column shows the actual screening recommendation issued by the trial, as final recommendations are subject to manual review by Screening Review Board in certain cases

Among non-PV carriers, use of the PRS in breast cancer risk stratification resulted in substantial changes in initial screening recommendations for individual participants. This was most conspicuous in women aged 40–49, where 14% (794/5752) had a different screening recommendation according to the BCSC-PRS risk score versus the BCSC risk score alone (Fig. 3A, Table S9). Given the starting age of screening in this age group is determined by risk, most changes affected whether the participant was recommended to start or defer screening. For instance, 11% (491/4466) of women that would have been recommended no screening under BCSC were recommended to start screening under BCSC-PRS. Only 7% (34/491) of these women would have been recommended to start screening in subsequent study years based on their BCSC risk score alone (Table S10). When we expanded the analysis to include women aged 40–49 with all versions of the PRS (*n* = 8608), we observed similar proportions of participants reassigned (Table S11).

For women aged 50–74, most recommendations were for biennial screening under both the BCSC-PRS and BCSC risk scores (Fig. 3B, Table S12). In this age group, the addition of PRS changed screening recommendations for 10% (801/7724) of participants. The PRS resulted in proportionally larger changes to the women receiving a recommendation for annual screening under BCSC alone, with 65% (436/675) reassigned to biennial and 21% (140/675) to yearly MRI plus mammogram. In women aged 50–74 with all versions of the PRS (*n* = 13,536), there were no substantial differences in proportions of participants reassigned (Table S13).

#### Net change in screening examinations

In women aged 40–49, BCSC-PRS recommendations resulted in 1 screen per 5 women/year compared to 1 screen per 6 women/year based on BCSC alone (Fig. S2). In women aged 50–74, BCSC-PRS and BCSC recommendations both resulted in 1 screen per 2 women per year. In both age groups, using BCSC-PRS risk led to higher numbers of twice-yearly and biennial screens and lower numbers of annual screens.

## Discussion

Our study outlines the first wave of results from our nationwide implementation of breast cancer PRS for personalized screening. In this analysis of 21,631 participants, we found our PRS had similar distributions by racial and ethnic group. As expected, higher PRS was associated with greater extent of family history and higher breast density. Most importantly, we found that incorporating the PRS into a clinical risk model changed the screening recommendations for a sizable group of participants of all ages without large changes to overall screening utilization. The availability of trial outcomes will allow us to better assess PRS discrimination and calibration by race.

Prior to the WISDOM trial, the impact of PRS on personalized screening had not been examined prospectively, and relatively little was known about the extent to which the PRS changes screening recommendations and affects screening utilization. We noted 14% of women aged 40–49 had a different screening recommendation after incorporating PRS into the BCSC model, with most of these changes related to the starting age of screening. Though the PRS resulted in slightly more women being recommended to start screening in their 40 s, there was a minimal increase in projected net screens per person/year. Among 50–74-year-olds, most of the changes affected women assigned to annual screening, and there was no change in projected net screens. In fact, compared with blanket annual screening starting age 40, which inherently recommends one screen per woman/year, the BCSC-PRS model recommends substantially fewer net screens such as an 80% reduction among those aged 40–49 and a 50% reduction among those aged 50–74. Indeed, if WISDOM were to find non-inferior advanced cancer incidence with risk-based screening, this would highlight the value of PRS in informing screening recommendations. Moreover, efforts are already underway to combine PRS with novel tools such as digital breast tomosynthesis image-based risk prediction, which has shown early promise in identifying women at high risk of breast cancer [17]. Such a personalized approach would be particularly impactful in women aged 40–49, for whom screening continues to generate the most controversy, and where women at lower risk who are properly informed are open to delay screening in their forties [18].

WISDOM is one of the first attempts to prospectively construct and deploy breast cancer PRSs in a diverse population. Thus, we were challenged with constructing PRSs for non-European ancestry populations with limited GWAS data, especially in the early stages of the trial. We based our approach on previous studies showing that European ancestry-derived PRSs can be tailored in East Asians and Latinas [19, 20] to have similar performance as in European women. Since we used the PRS together with the BCSC to generate an absolute risk estimate, we centered the PRS to 1 to minimize systematic over- or under-estimation of risk within each population, which may occur if the mean PRS deviates from 1 in a group of primarily unaffected (cancer-free) individuals. Overall, we found that our mean PRS approximated 1 across most groups, with several exceptions. The higher mean PRSs in women of other, mixed, or unknown race and the lower PRS in NH Black women may reflect small inaccuracies in centering PRS to the population, largely stemming from limitations in available SNP summary statistics and transferability of reference allele frequencies for these populations. The latter is particularly salient as we centered the PRSs based on allele frequencies from 1000 Genomes [21]. While we recognize that there are limits in generalizability between reference panels and our study population, we used reference allele frequencies because the allele frequencies in our study population could be subject to selection bias. For instance, WISDOM participants may be enriched for women with elevated risk, as suggested by the proportion of participants reporting a family history of breast cancer being higher than in other population-based studies [22]. This may have led the mean PRS in White women (the largest group) to be higher than 1.

Our findings also add to the understanding of the relationship between PRS and clinical risk factors. Few studies have been able to examine the relationship of PRS with detailed non-genetic and genetic risk factors in the screening (average-risk) population, with this knowledge having important implications for future risk modeling. Ours is among the first to examine the association between PRS and detailed family history, with our finding of highest PRS in women with first- and second-degree family history corroborating the PRS as a representation of inherited genetic risk for cancer. Other risk models account for the interaction between PRS and family history by attenuating the PRS effect size in those with family history of breast cancer. While our BCSC-PRS model did not include this adjustment, we expect the effect to be small for two reasons. First, prior studies have showed PRS and family history to have largely independent effects [8], suggesting the interaction is weak in magnitude. Second, the version of the BCSC model we used included first-degree family history as a binary term and did not account for the number of affected relatives, ages of onset, or second-degree family history. Thus, the model likely underestimates the familial component of risk, especially compared with pedigree-based models. Nevertheless, we plan to account for PRS interactions with family history and density in future versions of the BCSC-PRS model especially given the newest version of the BCSC model more extensively accounts for family history [23].

While we found a statistically significant correlation between the PRS and the BCSC score, the correlation was weak (*r*^2^ = 0.0041) and unlikely clinically important; we suspect the correlation is due to the association of the PRS with family history and density, both of which are contained in the BCSC score. Recent GWAS have shed light on the co-inheritance of breast density and breast cancer risk [24–26], thus the association between the PRS with density was expected. While a previous study found no correlation between the PRS and other clinical models, it examined a version of the model that did not include breast density [27].

A major strength of WISDOM is that we were able to prospectively generate and analyze PRS for 21,631 women with representation of major racial and ethnic groups in the USA. The population-based nature of our study contrasts with prior PRS studies, which have predominantly been case–control by design [8, 14], limited to women undergoing genetic testing [28], or done on archived biobank samples [29, 30]. Another strength of our study was the ability to examine PRS in the context of decision-making around screening.

Our findings should be interpreted considering several limitations. First, while we enhanced diversity during the trial through the inclusion of additional sites, our population ultimately had a lower percentage of Asian, Black, and Hispanic individuals compared with the overall US population [31]. Moreover, several racial and ethnic categories in our analysis contained relatively few individuals, which may limit our ability to definitively compare PRS distributions between these groups and others. Second, despite updating the PRS over the course of the trial to reflect the state of the PRS literature, we were unable to implement the most recent PRS with 313 SNPs (PRS313) [8]. The PRS313 was reported approximately 2 years into the trial and included genome-wide and non-genome-wide significant SNPs not included on our targeted capture panel. However, PRS constructed from genome-wide significant SNPs have comparable performance to expanded PRS such as the PRS313, with AUC differences of ~ 0.02 [8]. Another limitation related to the SNPs included on our panel was our inability to infer genetic ancestry, leading us to tailor PRS by self-reported race and ethnicity. This limitation is particularly salient to admixed populations such as Latinas, who can have vastly different degrees of Indigenous American, European, African, and Asian genetic ancestry [32], and the calibration of the PRS may be particularly affected in these groups. Once trial outcomes are available, we will be able to quantify the effects on performance of PRS by subgroup.

To address several of these limitations, the current iteration of WISDOM (2.0), which is actively enrolling, uses low pass whole genome sequencing. This will allow us to estimate genetic ancestry and employ multi-ancestry methods for PRS construction, for instance those that consider individual-level projections into principal component space [33–35]. Additionally, expanding the number of available SNPs will allow us to construct PRSs specifically developed for East Asian and African ancestry women [36–40], and PRSs for biological subtype [41, 42]. In WISDOM 2.0, the minimum age of eligibility is 30, and all participants will be able to self-select into risk-based versus annual screening. New trial sites with different catchment population demographics have been added.

## Conclusions

WISDOM represents an important first step in the implementation of PRS for risk-based screening. The early data presented here demonstrates qualitatively similar PRS distributions across demographic subgroups and expected associations with breast density and family history, which suggests our PRS should predict breast cancer. We found that PRS altered screening recommendations in small but substantial subgroups of women, without large changes in screening utilization. Our findings support the feasibility of implementing PRS to inform breast cancer screening in diverse populations and suggest a path for further refinement of personalized screening strategies at the population level.

## Supplementary Information

Additional file 1: Microsoft Word document containing Supplementary Methods.

Additional file 2: Microsoft Word document containing Tables S1–S13.

Additional file 3: Microsoft Word document containing Figs. S1 and S2.

## Data Availability

Data were generated by the authors but are not publicly available while trial follow-up remains underway. Upon completion of the trial and reporting of the primary outcomes, we plan to deposit genetic data in the database of Genotypes and Phenotypes (dbGaP). Summary statistics and phenotype data will also be available upon request to the study investigators, under terms set forth by the study’s institutional review board. We are committed to collaboration as a large multi-site trial and welcome future collaborators to get involved with WISDOM 2.0, which is now enrolling. Please contact the corresponding authors for further information on collaborating with the WISDOM team.

## Abbreviations

AUC: Area under the curve
BCSC: Breast Cancer Surveillance Consortium
BI-RADS: Breast Imaging Reporting and Data System
GWAS: Genome-wide association study
IQR: Interquartile range
NGS: Next-generation sequencing
NH: Non-Hispanic
OR: Odds ratio
PRS: Polygenic risk score
SD: Standard deviation
SNP: Single nucleotide polymorphism
WISDOM: Women Informed to Screen Depending On Measures of risk
