# Supplementary material for: Integrating breast cancer polygenic risk scores at scale in the WISDOM Study: a national randomized personalized screening trial
Source: Genome Med. 2025 Aug 28;17:97. doi: 10.1186/s13073-025-01524-7 (PMC12395744; doi:10.1186/s13073-025-01524-7)
Supplement: Supplementary file 1 — Additional file 1: Microsoft Word document containing Supplementary Methods. [file 13073_2025_1524_MOESM1_ESM.docx]

**SUPPLEMENTARY METHODS**

**Genotyping**

DNA was extracted from saliva, followed by target enrichment with an automated hybrid capture procedure using Agilent’s SureSelect XT probes (Santa Clara, CA, USA). Sequencing and SNP genotyping were performed using Illumina’s NextSeq 500/550 (San Diego, CA, USA) software, and reads were aligned to GRCh37.p12 human genome reference. Single nucleotide polymorphisms (SNPs) were chosen for this NGS-target enrichment assay based on prior genome wide association studies (GWAS), (**Supplemental Table 1**). The bioinformatics pipeline utilized Genome-Analysis Toolkit (GATK) v3.4 as well as complementary algorithms including DeepVariant (v0.10.0) (1) and Scalpel (v0.5.3) (2). Quality control and assurance included removal of all duplicate and low-quality reads. Two positive controls with known variants were included in each batch. Batch effects were addressed by normalizing the coverage of samples against a reference set of samples sequenced in the same batch. In addition, only samples with a minimum of 223 out of 228 SNPs at a coverage of at least 20X were eligible for PRS construction. Potential false calls were identified using a machine learning model developed internally at Color supplemented with orthogonal confirmation with Sanger sequencing (3). Color is certified by Clinical Laboratory Improvement Amendments (05D2081492) and accredited by the College of American Pathologists (8975161), which requires rigorous quality control and quality assurance measures.

**Construction of PRS by race and ethnicity groups**

Generating a polygenic risk score (PRS) for diverse participants in a large, national screening trial involved several methodological considerations. First, given the adaptive nature of the WISDOM Study, we sought to keep pace with the discovery of new SNPs associated with breast cancer that occurred after the trial started by iteratively generating new versions of the PRS as more GWAS results became available. Second, we elected to construct separate PRSs for four major racial and ethnic groups based on self-report, including Asian (Asian plus Native Hawaiian/Asian Pacific Islander), Black, Hispanic (for any participant indicating Hispanic ethnicity, regardless of race), and White. We selected these groups because they were anticipated to represent the most common racial and ethnic groups enrolled in the WISDOM trial and because the baseline risk model which we used, the Breast Cancer Surveillance Consortium (BCSC) model, includes risk estimates for these groups but not others. Given the limited GWAS data available for those self-reporting “Other,” “Mixed Race,” Native American/Alaskan Native, and Native Hawaiian populations, we calculated their PRS using parameters for White women. The number of SNPs in the PRS varied by race and ethnicity group ranging from 118 to 126. Relevant GWAS studies for creating the study PRSs are shown in **Supplemental Table 1** for each respective racial and ethnic group.

To reduce the likelihood of allele selection with differential effects among those with non-White genetic ancestry, we tested the performance of SNPs discovered in primarily European GWAS (4-6) in publicly available external datasets with participants with non-European ancestry. For African Americans, we used the ROOT (7) dataset and a consortium of datasets reported by Chen et al. in 2013 (8). For East Asian ancestry, we used a series of published GWAS studies (9-15) and for Hispanic/Latina women we used our own previously published GWAS studies (16, 17). A SNP was identified as directionally consistent if the risk alleles were the same in each population and/or if the odds ratio (OR) identified in Europeans fell within the 95% confidence interval of the OR for the same allele in the other ancestry population. SNPs in linkage disequilibrium were removed (r2 >0.3), and if multiple SNPs at a single locus were reported, the SNP most strongly associated at that locus was selected.

We calculated the PRS as the product of the likelihood ratios (LRs) for each included SNP, with each LR being a function of the number of risk alleles present, the risk allele frequency, and associated odds ratio (OR) for each SNP (18, 19). The LR approach has the advantage that, if allele frequencies are specified correctly, the mean PRS in the population should be ~1. For each of four major racial or ethnic groups, we used allele frequencies for analogous reference populations in 1000 Genomes (20): African (AFR), East Asian (EAS), Ad-Mixed American (AMR), and European (EUR). We used published ORs from the Breast Cancer Association Consortium GWAS for each SNP (6). For SNPs that were discovered in non-European populations, we used the ORs from the respective discovery GWAS. Our calculation assumes that SNPs are in Hardy-Weinberg equilibrium, with independent, log-additive effects. This assumption was tested post-hoc in self-identified White participants (the largest racial/ethnic group), and we found no evidence of departure from Hardy-Weinberg equilibrium after Bonferroni correction. The incorporation of population-specific allele frequencies is expected to center the mean PRS to approximately 1 in cancer-free individuals within each respective population. Further details on our PRS calculation method have been previously described (18, 21). To generate the BCSC-PRS risk estimate, we combined the PRS with the BCSC 5-year risk estimate in a Bayesian manner, as previously described (18). In short, we used the participant’s five-year risk of breast cancer (D^+^) from the BCSC score as the pre-test probability (P_prior_), and generated participant-level breast cancer risk LRs as the product of the individual genotype (G_i_) SNP LR’s according to the below formula:

$$P\left( D^{+} \right| G_{i})=\frac{P_{prior}*LR}{P_{prior}*\left( LR-1 \right)+1}$$

**WISDOM Study power calculation**

To calculate power for the overall WISDOM Study, we first assumed in the risk-based screening arm that the proportion of participants in the biennial, annual, and high-risk (every 6 month) screening groups would be 40.4%, 28.2%, and 2.5% respectively. The remaining 28.9% would therefore receive a screening recommendation corresponding to no screening during the trial. Next, we approximated the overall rate of Stage IIB cancers to be about 0.05% per year and assumed that the hazard for Stage IIB cancers would increase along with risk group. We therefore projected the annual hazard for Stage IIB cancer to be 0.020% in the no screening risk group, 0.041% in the biennial screening group, 0.077% in the annual screening group, and 0.28% in the high-risk group. Using projections of WISDOM enrollment of 4,500 randomized participants per year, an enrollment period of seven years, and a maximum follow-up of nine years will result in about 32,000 person-years of exposure time in each arm (assuming a loss to follow-up rate of 20%). This allows us to achieve 76% power to show noninferiority of risk-based screening compared to annual screening with respect to rate of diagnosed stage ≥ IIB cancers (using a non-inferiority delta of 0.0005 and assuming no difference in the rate of stage ≥ IIB cancers between the arms).

**References**

1. Poplin R, Chang P-C, Alexander D, Schwartz S, Colthurst T, Ku A, et al. A universal SNP and small-indel variant caller using deep neural networks. Nature biotechnology. 2018;36(10):983-7.

2. Fang H, Bergmann EA, Arora K, Vacic V, Zody MC, Iossifov I, et al. Indel variant analysis of short-read sequencing data with Scalpel. Nature protocols. 2016;11(12):2529-48.

3. van den Akker J, Mishne G, Zimmer AD, Zhou AY. A machine learning model to determine the accuracy of variant calls in capture-based next generation sequencing. BMC Genomics. 2018;19(1):263.

4. Michailidou K, Hall P, Gonzalez-Neira A, Ghoussaini M, Dennis J, Milne RL, et al. Large-scale genotyping identifies 41 new loci associated with breast cancer risk. Nat Genet. 2013;45(4):353-61, 61e1-2.

5. Michailidou K, Beesley J, Lindstrom S, Canisius S, Dennis J, Lush MJ, et al. Genome-wide association analysis of more than 120,000 individuals identifies 15 new susceptibility loci for breast cancer. Nat Genet. 2015;47(4):373-80.

6. Michailidou K, Lindström S, Dennis J, Beesley J, Hui S, Kar S, et al. Association analysis identifies 65 new breast cancer risk loci. Nature. 2017;551(7678):92-4.

7. Wang S, Qian F, Zheng Y, Ogundiran T, Ojengbede O, Zheng W, et al. Genetic variants demonstrating flip-flop phenomenon and breast cancer risk prediction among women of African ancestry. Breast Cancer Res Treat. 2018;168(3):703-12.

8. Chen F, Chen GK, Stram DO, Millikan RC, Ambrosone CB, John EM, et al. A genome-wide association study of breast cancer in women of African ancestry. Human genetics. 2013;132(1):39-48.

9. Cai Q, Long J, Lu W, Qu S, Wen W, Kang D, et al. Genome-wide association study identifies breast cancer risk variant at 10q21.2: results from the Asia Breast Cancer Consortium. Human molecular genetics. 2011;20(24):4991-9.

10. Cai Q, Zhang B, Sung H, Low SK, Kweon SS, Lu W, et al. Genome-wide association analysis in East Asians identifies breast cancer susceptibility loci at 1q32.1, 5q14.3 and 15q26.1. Nat Genet. 2014;46(8):886-90.

11. Egan KM, Cai Q, Shu XO, Jin F, Zhu TL, Dai Q, et al. Genetic polymorphisms in GSTM1, GSTP1, and GSTT1 and the risk for breast cancer: results from the Shanghai Breast Cancer Study and meta-analysis. Cancer Epidemiol Biomarkers Prev. 2004;13(2):197-204.

12. Kim HC, Lee JY, Sung H, Choi JY, Park SK, Lee KM, et al. A genome-wide association study identifies a breast cancer risk variant in ERBB4 at 2q34: results from the Seoul Breast Cancer Study. Breast Cancer Res. 2012;14(2):R56.

13. Long J, Cai Q, Sung H, Shi J, Zhang B, Choi JY, et al. Genome-wide association study in east Asians identifies novel susceptibility loci for breast cancer. PLoS Genet. 2012;8(2):e1002532.

14. Low SK, Takahashi A, Ashikawa K, Inazawa J, Miki Y, Kubo M, et al. Genome-wide association study of breast cancer in the Japanese population. PLoS One. 2013;8(10):e76463.

15. Zheng W, Long J, Gao YT, Li C, Zheng Y, Xiang YB, et al. Genome-wide association study identifies a new breast cancer susceptibility locus at 6q25.1. Nat Genet. 2009;41(3):324-8.

16. Fejerman L, Ahmadiyeh N, Hu D, Huntsman S, Beckman KB, Caswell JL, et al. Genome-wide association study of breast cancer in Latinas identifies novel protective variants on 6q25. Nat Commun. 2014;5:5260.

17. Hoffman J, Fejerman L, Hu D, Huntsman S, Li M, John EM, et al. Identification of novel common breast cancer risk variants at the 6q25 locus among Latinas. Breast Cancer Res. 2019;21(1):3.

18. Shieh Y, Hu D, Ma L, Huntsman S, Gard CC, Leung JW, et al. Breast cancer risk prediction using a clinical risk model and polygenic risk score. Breast cancer research and treatment. 2016;159:513-25.

19. Lu Q, Elston RC. Using the optimal receiver operating characteristic curve to design a predictive genetic test, exemplified with type 2 diabetes. Am J Hum Genet. 2008;82(3):641-51.

20. Auton A, Brooks LD, Durbin RM, Garrison EP, Kang HM, Korbel JO, et al. A global reference for human genetic variation. Nature. 2015;526(7571):68-74.

21. Ziv E, Tice JA, Sprague B, Vachon CM, Cummings SR, Kerlikowske K. Using Breast Cancer Risk Associated Polymorphisms to Identify Women for Breast Cancer Chemoprevention. PLoS One. 2017;12(1):e0168601.
