# Supplementary material for: Integrating breast cancer polygenic risk scores at scale in the WISDOM Study: a national randomized personalized screening trial
Source: Genome Med. 2025 Aug 28;17:97. doi: 10.1186/s13073-025-01524-7 (PMC12395744; doi:10.1186/s13073-025-01524-7)
Supplement: Supplementary file 2 — Additional file 2: Microsoft Word document containing Tables S1–S13. [file 13073_2025_1524_MOESM2_ESM.docx]

**Table S1:** Summary of sources for race and ethnicity-specific polygenic risk scores

| **Racial or ethnic group for PRS**^a^ | **Number of SNPs included in PRS** | **Reference population for allele frequencies**^b^ | **Reference studies for external validation** |
| --- | --- | --- | --- |
| Non-Hispanic Asian | 125 | EAS | Egan et al. (2004)(1)  Zheng et al. (2009)(2)  Cai et al. (2011)(3)  Long et al. (2012)(4)  Kim et al. (2012)(5)  Low et al. (2013)(6)  Cai et al. (2014)(7) |
| Non-Hispanic Black | 118 | AFR | Chen et al. (2013)(8)  Wang et al. (2018)(9) |
| Hispanic | 126 | AMR | Fejerman et al. (2014)(10)  Hoffman et al. (2019)(11) |
| Non-Hispanic White | 126 | EUR | Michailidou et al. (2017)(12) |

| *^a^* PRS: Polygenic risk score |
| --- |
| *^b^* EAS: East Asian; AFR: African; AMR: Ad-Mixed American; EUR: European |

**Table S2:** Risk thresholds table showing the criteria for each screening recommendation

| BCSC-PRS  Screening recommendation | Age 40-49 | Age ≥ 50 | |
| --- | --- | --- | --- |
| No screening at this time | **5-year risk** < 1.3%^a^ | **-** | |
| Stop Screening | **-** | Age 75+ AND 5-year risk <1.3%^a,b^ | |
| Biennial mammogram | 5-year risk ≥ 1.3%  **AND**  5-years risk < 97.5^th^% by age | 5-years risk < 97.5^th^% by age | |
| Annual mammogram | Extremely dense breasts (BIRADS d) on prior mammogram | - | |
|  | - | Age 65+: 5-year risk ≥ 6%^c^ **AND** non-pathogenic variant carrier **AND** BIRADS density a or b or BIRADS density c or d **AND** lifetime risk <20% | |
|  | 5-year risk ≥ 97.5^th^% by age | | |
|  | Carriers of *ATM* or *CHEK2* **WITHOUT** a positive family history of breast cancer^d^ | | |
|  | Biopsy with atypia **AND** first-degree family history of breast cancer^e^ **AND** no chemoprevention modifier | | |
| Annual mammogram + adjunctive MRI (screen every six months) | Age 40-64:  5-year risk ≥ 6%^c^ |  | Age 65+:  5-year risk ≥ 6%^c^ **AND** BIRADS density c or d **AND** a lifetime risk ≥ 20%^f^ |
|  | *BRCA1/2, TP53, PTEN, STK11, CDH1, PALB2* mutation | | |
|  | *ATM* or *CHEK2* **WITH** a positive family history of breast cancer^d^ | | |
|  | History of chest wall radiation before age 35 | | |

KEY:

^a^Average risk of a 50-year-old woman. Participants are assigned an age to start screening when their risk reaches that of the average 50 year old; therefore some are assigned to screen at an age under 50.

^b^ In addition, stop screening if age 70+ AND 5-year risk < 2.2% AND if 50% chance of mortality (based on ePrognosis)

^c^Average risk of BRCA carrier

^d^If positive family history (e.g. first degree relative diagnosed < age 50; first degree relative plus second degree relative age < 50; or two second degree relatives one age < 50) and *ATM*/*CHEK2* carrier, will receive annual mammography plus adjunctive MRI

^e^First degree family history of breast cancer only (e.g. mother, sister, daughter)

^f^BCSC+PRS lifetime risk OR Tyrer-Cuzick lifetime risk

**Table S3:** Characteristics of Screening Review Board

| **Composition** | At least one study physician lead, genetics lead, and Breast Health Specialist (genetic counselor) |
| --- | --- |
| **Meeting frequency** | Monthly |
| **Criteria triggering Screening Review Board discussion** | Participants younger than 60 with a screening assignment of No screening, biennial screening, or annual screening who have the following family history criteria:^a^   - 2 FDR with breast cancer AND one diagnosed at age 50 or younger - 2 or more FDR/SDR with combination of breast and ovarian cancer regardless of age - 2 or more FDR/SDR with breast cancer, with youngest <40 years of age at diagnosis - 3 or more FDR/SDR with breast cancer regardless of age - Male breast cancer - 1 FDR and 1 SDR with breast cancer AND BOTH diagnosed 50 or younger - 1 FDR diagnosed 50 or younger AND participant is under 50 years old |
| **Decision** | Confirm or modify current screening assignment |

^a^Family history must be within the same bloodline, meaning the relatives diagnosed all must be within the same maternal/paternal side. FDR = first degree relative, SDR = second degree relative.

**Table S4:** Characteristics of risk-based arm participants included and excluded from the analysis

| **Characteristic** | **Included in analysis**  **N = 21,631***^1^* | **Excluded from analysis**  **N = 8,504** | **Standardized mean difference (95% confidence interval)** |
| --- | --- | --- | --- |
| **Age** | 53 (45, 62) | 52 (45, 60) | -0.08 (-0.10 to -0.05) |
| **Race or Ethnicity**^2^ |  |  | 0.21 (0.19 to 0.24) |
| Hispanic | 1,932 (8.9%) | 989 (12%) |  |
| NH American Indian or Alaska Native | 44 (0.2%) | 39 (0.5%) |  |
| NH Asian/NH Hawaiian, Pacific Islander | 1,029 (4.8%) | 472 (5.5%) |  |
| NH Black | 969 (4.5%) | 685 (8.1%) |  |
| NH more than one race/Other race/Unknown race | 875 (4.0%) | 437 (5.1%) |  |
| NH White | 16,782 (78%) | 5,882 (69%) |  |
| **Family history of breast cancer** |  |  | 0.09 (0.06 to 0.11) |
| No family history | 10,433 (48%) | 4,397 (52%) |  |
| Only second degree relative | 5,302 (25%) | 2,096 (25%) |  |
| Only first degree relative | 3,369 (16%) | 1,192 (14%) |  |
| Both first and second degree relative | 2,527 (12%) | 819 (9.6%) |  |
| **Pathogenic variant carrier** | 665 (3.1%) | 42 (0.5%) | -0.20 (-0.22 to -0.17) |
| **Breast density (BI-RADS category)** |  |  | -0.09 (-0.12 to -0.06) |
| 1. Almost entirely fatty | 1,009 (4.7%) | 372 (6.1%) |  |
| 1. Scattered areas of fibroglandular density | 7,565 (35%) | 2,632 (43%) |  |
| 1. Heterogeneously dense | 8,581 (40%) | 2,633 (43%) |  |
| 1. Extremely dense | 1,876 (8.7%) | 491 (8.0%) |  |
| Unknown | 2,600 (12%) | 2,376 |  |
| *^1^* Median (IQR); n (%) | | | |
| *^2^* NH: Non-Hispanic | | | |

**Table S5:** Polygenic risk score by race and ethnicity categories

|  | **Overall**  N = 21,631 | **Hispanic**  N = 1,932 | **NH**^1^ **American Indian or Alaska Native**  N = 44 | **NH Asian/NH Hawaiian, Pacific Islander**  N = 1,029 | **NH Black**  N = 969 | **NH more than one race/Other race/Unknown race**  N = 875 | **NH White**  N = 16,782 |
| --- | --- | --- | --- | --- | --- | --- | --- |
| **PRS**^2^ |  |  |  |  |  |  |  |
| Median (IQR) | 0.92 (0.67, 1.28) | 0.88 (0.66, 1.24) | 0.92 (0.72, 1.28) | 0.86 (0.64, 1.19) | 0.82 (0.59, 1.19) | 0.97 (0.70, 1.37) | 0.93 (0.68, 1.29) |
| Mean (SD) | 1.04 (0.54) | 1.00 (0.52) | 1.01 (0.41) | 0.99 (0.53) | 0.96 (0.55) | 1.11 (0.57) | 1.05 (0.54) |
| *^1^* NH: Non-Hispanic | | | | | | | |
| *^2^* PRS: polygenic risk score; IQR: Interquartile range; SD: Standard deviation | | | | | | | |

**Table S6:** Quintile distribution of polygenic risk score by race and ethnicity categories

|  | **Overall**  N = 21,631*^1^* | **Hispanic**  N = 1,932*^1^* | **NH**^2^ **American Indian or Alaska Native**  N = 44*^1^* | **NH Asian/NH Hawaiian, Pacific Islander**  N = 1,029*^1^* | **NH Black**  N = 969*^1^* | **NH more than one race/Other race/Unknown race**  N = 875*^1^* | **NH White**  N = 16,782*^1^* | **p-value***^3^* |
| --- | --- | --- | --- | --- | --- | --- | --- | --- |
| **PRS**^4^  **Quintiles** |  |  |  |  |  |  |  | **<0.001** |
| 1 | 4,327 (20%) | 419 (22%) | 8 (18%) | 242 (24%) | 281 (29%) | 148 (17%) | 3,229 (19%) |  |
| 2 | 4,326 (20%) | 407 (21%) | 9 (20%) | 220 (21%) | 197 (20%) | 171 (20%) | 3,322 (20%) |  |
| 3 | 4,326 (20%) | 391 (20%) | 10 (23%) | 211 (21%) | 172 (18%) | 162 (19%) | 3,380 (20%) |  |
| 4 | 4,326 (20%) | 358 (19%) | 9 (20%) | 183 (18%) | 156 (16%) | 179 (20%) | 3,441 (21%) |  |
| 5 | 4,326 (20%) | 357 (18%) | 8 (18%) | 173 (17%) | 163 (17%) | 215 (25%) | 3,410 (20%) |  |
| *^1^*n (%) | | | | | | | | |
| *^2^*NH: Non-Hispanic | | | | | | | | |
| *^3^*Pearson's Chi-squared test | | | | | | | | |
| *^4^*PRS: polygenic risk score; Quintiles: overall study population divided into quintiles of PRS, with the lowest scores in quintile 1 and the highest scores in quintile 5 | | | | | | | | |

**Table S7:** Polygenic risk score by detailed family history of breast cancer

| **Characteristic** | **Overall**  N = 20,594 | **No relatives with BC^1^**  N = 9,843 | **1 relative with BC, diagnosed at age 50 and above (or unknown)**  N = 4,899 | **1 relative with BC, diagnosed before age 50**  N = 1,571 | **≥2 relatives with BC, diagnosed at age 50 and above (or unknown)**  N = 1,981 | **≥2 relatives with BC, ≥1 diagnosed before age 50**  N = 1,539 | **Unknown family history**  N = 761 | **p-value***^2^* |
| --- | --- | --- | --- | --- | --- | --- | --- | --- |
| **PRS^3^** |  |  |  |  |  |  |  | **<0.001** |
| Median (Q1, Q3) | 0.92 (0.67, 1.27) | 0.88 (0.65, 1.23) | 0.93 (0.67, 1.28) | 0.95 (0.69, 1.30) | 0.98 (0.73, 1.36) | 1.00 (0.72, 1.40) | 0.93 (0.69, 1.37) |  |
| Mean (SD) | 1.04 (0.53) | 0.99 (0.50) | 1.04 (0.55) | 1.06 (0.54) | 1.11 (0.57) | 1.13 (0.58) | 1.08 (0.54) |  |
| *^1^*BC: breast cancer | | | | | | | | |
| *^2^*Kruskal-Wallis rank sum test | | | | | | | | |
| ^3^PRS: polygenic risk score | | | | | | | | |

**Table S8:** Polygenic risk score by breast density

|  |  | **BIRADS Density Category**^1^ | | | |  |
| --- | --- | --- | --- | --- | --- | --- |
| **PRS**^3^ | **Overall**  N = 19,031 | **a, almost entirely fatty**  N = 1,009 | **b, scattered areas of fibroglandular density**  N = 7,565 | **c, heterogeneously dense**  N = 8,581 | **d, extremely dense**  N = 1,876 | **p-value***^2^* |
| Median (IQR) | 0.93 (0.68, 1.29) | 0.87 (0.64, 1.21) | 0.92 (0.66, 1.28) | 0.94 (0.69, 1.30) | 0.96 (0.71, 1.31) | **<0.001** |
| Mean (SD) | 1.05 (0.54) | 0.98 (0.48) | 1.03 (0.53) | 1.06 (0.56) | 1.08 (0.57) | **<0.001** |
| *^1^*BIRADS: Breast Imaging Reporting and Data System | | | | | | |
| *^2^*Kruskal-Wallis rank sum test; additional test of trend p<0.001 | | | | | | |
| *^3^*PRS: Polygenic risk score; IQR: Interquartile range; SD: Standard deviation | | | | | | |

**Table S9:** Cross-tabulation of screening assignments by BCSC vs BCSC-PRS, women aged 40-49 whose assignments were based on most recent version of PRS

|  | **Assignment based on BCSC-PRS**^1^ | | | | |
| --- | --- | --- | --- | --- | --- |
| **Assignment based on BCSC** | **6mo based on risk**  N = 13*^2^* | **Annual based on density**  N = 592*^2^* | **Annual based on risk**  N = 173*^2^* | **Biennial**  N = 817*^2^* | **No screening**  N = 4,157*^2^* |
| 6mo based on risk | 1 (100%) | 0 (0%) | 0 (0%) | 0 (0%) | 0 (0%) |
| Annual based on density | 5 (0.8%) | 592 (99%) | 0 (0%) | 0 (0%) | 0 (0%) |
| Annual based on risk | 7 (3.8%) | 0 (0%) | 73 (40%) | 63 (34%) | 40 (22%) |
| Biennial | 0 (0%) | 0 (0%) | 46 (9.1%) | 317 (63%) | 142 (28%) |
| No screening | 0 (0%) | 0 (0%) | 54 (1.2%) | 437 (9.8%) | 3,975 (89%) |
| *^1^*BCSC: Breast Cancer Surveillance Consortium; PRS: polygenic risk score; BCSC-PRS: Study risk tool integrating PRS into the BCSC | | | | | |
| *^2^*n (%) | | | | | |

**Table S10:** Anticipated screening assignments based on BCSC risk score in Study Years 2-3 for risk-based arm participants aged 40-49 recommended to start screening in Year 1 due to PRS

|  | **All^1^**  N = 491 |
| --- | --- |
| Start screening in Year 2 due to BCSC^2^ risk^3^ | 17 (3.5%) |
| Start screening in Year 3 due to BCSC^2^ risk^3^ | 17 (3.5%) |
| **Total** | 34 (7.0%) |
| ^1^n (%) | |
| ^2^BCSC: Breast Cancer Screening Consortium | |
| ^3^Excludes participants if they were recommended to start screening due to turning 50 as this is recommendation is independent of BCSC risk | |

**Table S11.** Cross-tabulation sensitivity analysis of screening assignments by BCSC vs BCSC-PRS, women aged 40-49 whose assignments were based on any version of the PRS

|  | **Assignment based on BCSC-PRS**^1^ | | | | |
| --- | --- | --- | --- | --- | --- |
| **Assignment based on BCSC** | **6mo based on risk**  N = 34*^2^* | **Annual based on density**  N = 1,002*^2^* | **Annual based on risk**  N = 290*^2^* | **Biennial**  N = 1,268*^2^* | **No screening**  N = 6,014*^2^* |
| 6mo based on risk | 2 (67%) | 1 (33%) | 0 (0%) | 0 (0%) | 0 (0%) |
| Annual based on density | 11 (1.1%) | 1,001 (99%) | 0 (0%) | 0 (0%) | 0 (0%) |
| Annual based on risk | 18 (6.1%) | 0 (0%) | 102 (34%) | 108 (36%) | 69 (23%) |
| Biennial | 3 (0.4%) | 0 (0%) | 78 (9.7%) | 465 (58%) | 260 (32%) |
| No screening | 0 (0%) | 0 (0%) | 110 (1.7%) | 695 (11%) | 5,685 (88%) |
| *^1^*BCSC: Breast Cancer Surveillance Consortium; PRS: polygenic risk score; BCSC-PRS: Study risk tool integrating PRS into the BCSC | | | | | |
| *^2^*n (%) | | | | | |

**Table S12:** Cross-tabulation of screening assignments by BCSC vs BCSC-PRS, women aged 50-74 whose assignments were based on most recent version of PRS

|  | **Assignment based on BCSC-PRS**^1^ | | | |
| --- | --- | --- | --- | --- |
| **Assignment based on BCSC** | **6mo based on risk**  N = 234*^2^* | **Annual based on risk**  N = 237*^2^* | **Biennial**  N = 7,245*^2^* | **Stop screen low risk**  N = 8*^2^* |
| 6mo based on risk | 21 (57%) | 6 (16%) | 10 (27%) | 0 (0%) |
| Annual based on risk | 140 (21%) | 99 (15%) | 436 (65%) | 0 (0%) |
| Biennial | 73 (1.0%) | 132 (1.9%) | 6,799 (97%) | 4 (<0.1%) |
| Stop screen low risk | 0 (0%) | 0 (0%) | 0 (0%) | 4 (100%) |
| *^1^*BCSC: Breast Cancer Surveillance Consortium; PRS: polygenic risk score; BCSC-PRS: Study risk tool integrating PRS into the BCSC | | | | |
| *^2^*n (%) | | | | |

**Table S13:** Cross-tabulation sensitivity analysis of screening assignments by BCSC vs BCSC-PRS, women aged 50-74 whose assignments were based on any version of the PRS

|  | **Assignment based on BCSC-PRS**^1^ | | | |
| --- | --- | --- | --- | --- |
| **Assignment based on BCSC** | **6mo based on risk**  N = 476*^2^* | **Annual based on risk**  N = 479*^2^* | **Biennial**  N = 12,442*^2^* | **Stop screen low risk**  N = 139*^2^* |
| 6mo based on risk | 39 (60%) | 9 (14%) | 17 (26%) | 0 (0%) |
| Annual based on risk | 247 (20%) | 183 (14%) | 832 (66%) | 3 (0.2%) |
| Biennial | 190 (1.6%) | 287 (2.4%) | 11,582 (95%) | 117 (1.0%) |
| Stop screen low risk | 0 (0%) | 0 (0%) | 11 (37%) | 19 (63%) |
| *^1^*BCSC: Breast Cancer Surveillance Consortium; PRS: polygenic risk score; BCSC-PRS: Study risk tool integrating PRS into the BCSC | | | | |
| *^2^*n (%) | | | | |

**REFERENCES**

1. Egan KM, Cai Q, Shu XO, Jin F, Zhu TL, Dai Q, et al. Genetic polymorphisms in GSTM1, GSTP1, and GSTT1 and the risk for breast cancer: results from the Shanghai Breast Cancer Study and meta-analysis. Cancer Epidemiol Biomarkers Prev. 2004;13(2):197-204.

2. Zheng W, Long J, Gao YT, Li C, Zheng Y, Xiang YB, et al. Genome-wide association study identifies a new breast cancer susceptibility locus at 6q25.1. Nat Genet. 2009;41(3):324-8.

3. Cai Q, Long J, Lu W, Qu S, Wen W, Kang D, et al. Genome-wide association study identifies breast cancer risk variant at 10q21.2: results from the Asia Breast Cancer Consortium. Human molecular genetics. 2011;20(24):4991-9.

4. Long J, Cai Q, Sung H, Shi J, Zhang B, Choi JY, et al. Genome-wide association study in east Asians identifies novel susceptibility loci for breast cancer. PLoS Genet. 2012;8(2):e1002532.

5. Kim HC, Lee JY, Sung H, Choi JY, Park SK, Lee KM, et al. A genome-wide association study identifies a breast cancer risk variant in ERBB4 at 2q34: results from the Seoul Breast Cancer Study. Breast Cancer Res. 2012;14(2):R56.

6. Low SK, Takahashi A, Ashikawa K, Inazawa J, Miki Y, Kubo M, et al. Genome-wide association study of breast cancer in the Japanese population. PLoS One. 2013;8(10):e76463.

7. Cai Q, Zhang B, Sung H, Low SK, Kweon SS, Lu W, et al. Genome-wide association analysis in East Asians identifies breast cancer susceptibility loci at 1q32.1, 5q14.3 and 15q26.1. Nat Genet. 2014;46(8):886-90.

8. Chen F, Chen GK, Stram DO, Millikan RC, Ambrosone CB, John EM, et al. A genome-wide association study of breast cancer in women of African ancestry. Human genetics. 2013;132(1):39-48.

9. Wang S, Qian F, Zheng Y, Ogundiran T, Ojengbede O, Zheng W, et al. Genetic variants demonstrating flip-flop phenomenon and breast cancer risk prediction among women of African ancestry. Breast Cancer Res Treat. 2018;168(3):703-12.

10. Fejerman L, Ahmadiyeh N, Hu D, Huntsman S, Beckman KB, Caswell JL, et al. Genome-wide association study of breast cancer in Latinas identifies novel protective variants on 6q25. Nat Commun. 2014;5:5260.

11. Hoffman J, Fejerman L, Hu D, Huntsman S, Li M, John EM, et al. Identification of novel common breast cancer risk variants at the 6q25 locus among Latinas. Breast Cancer Res. 2019;21(1):3.

12. Michailidou K, Lindström S, Dennis J, Beesley J, Hui S, Kar S, et al. Association analysis identifies 65 new breast cancer risk loci. Nature. 2017;551(7678):92-4.
