# Supplementary material for: Integrating breast cancer polygenic risk scores at scale in the WISDOM Study: a national randomized personalized screening trial
Source: Genome Med. 2025 Aug 28;17:97. doi: 10.1186/s13073-025-01524-7 (PMC12395744; doi:10.1186/s13073-025-01524-7)

**Figure S1. Scatter plot of PRS versus BCSC 5-year risk score**

Scatter plot of included participants with calculated polygenic risk score reported on log-scale on the x-axis and the Breast Cancer Surveillance Consortium (BCSC) score on the y-axis. IQR: Interquartile range is indicated by dotted blue lines. Pearson’s correlation coefficient r^2^ = 0.0041 (p < 0.001).


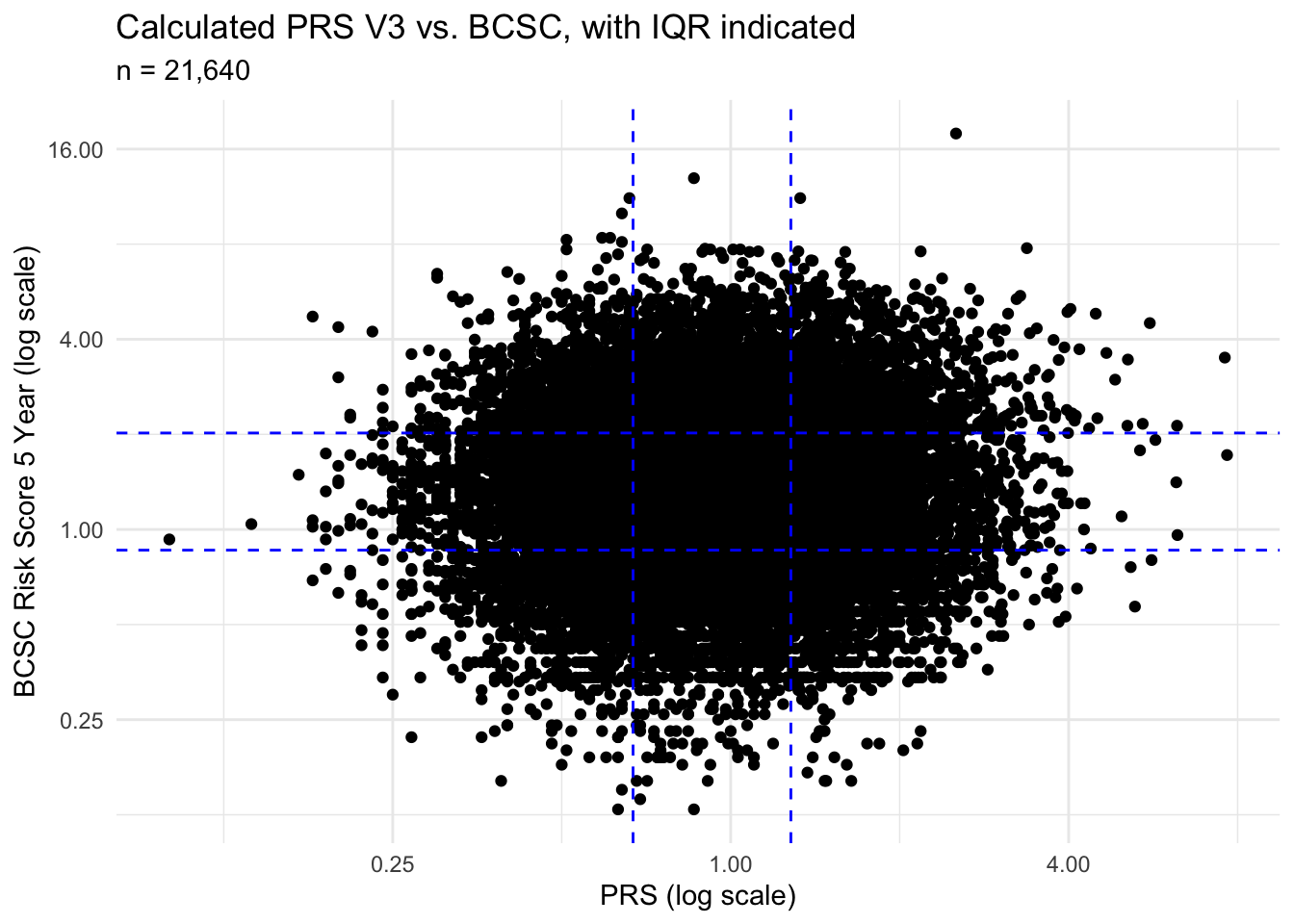


**Figure S2. Net changes in screening recommendations comparing BCSC to BCSC-PRS**

Waterfall plot of projected net change in screening encounters in (A) participants aged 40-49 and (B) participants aged 50-74 at study entry. The change in absolute number of screening encounters is calculated by subtracting Breast Cancer Surveillance Consortium (BCSC) recommended encounters from those after integrating polygenic risk scores (BCSC-PRS).


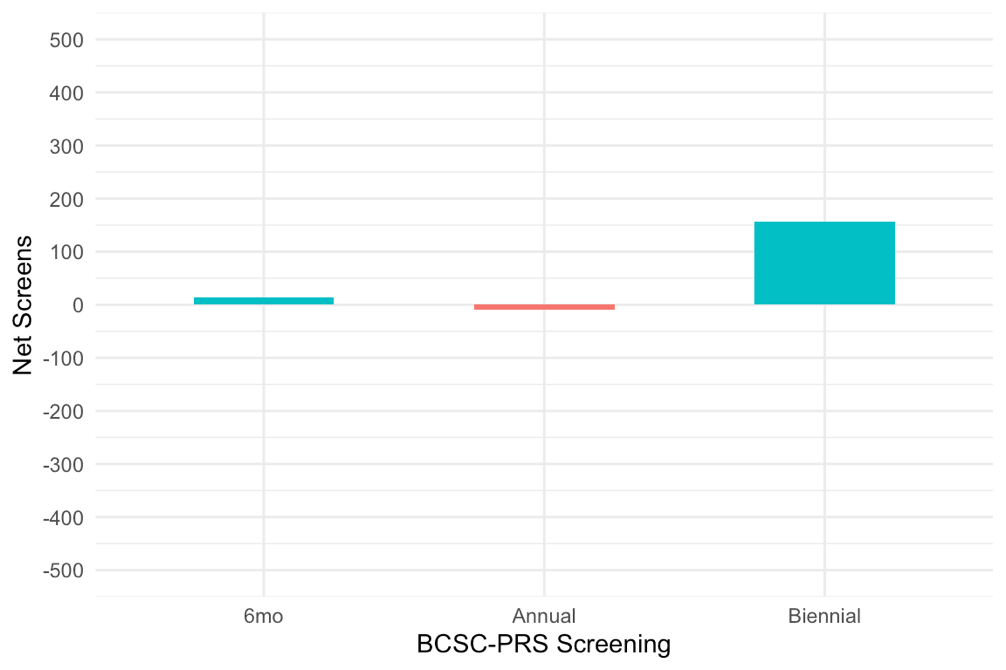


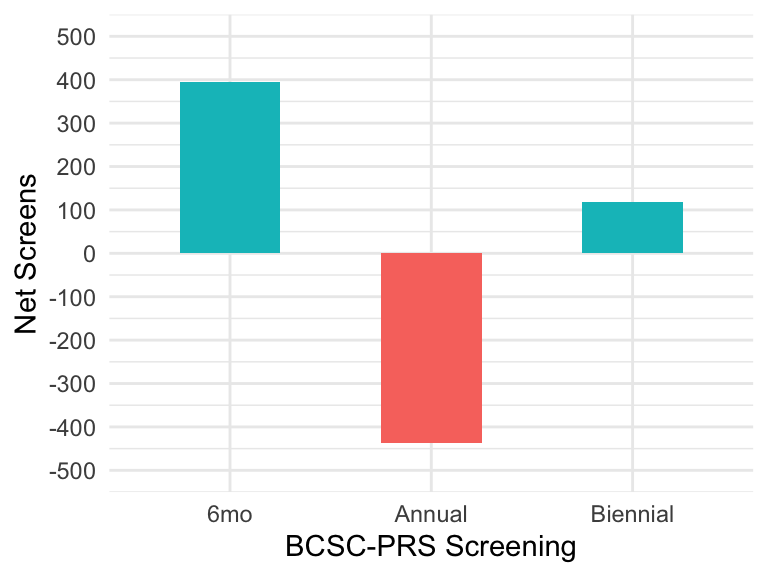

Supplement: Supplementary file 3 — Additional file 3: Microsoft Word document containing Figs. S1 and S2. [file 13073_2025_1524_MOESM3_ESM.docx]
